# Pregnant patient with Xp11.2/transcription factor E3 translocation renal cell carcinoma: a case report and literature review

**DOI:** 10.3389/fonc.2024.1388880

**Published:** 2024-06-26

**Authors:** Yanchen Wang, Xiaoyan Guo, Zhe Meng, Yong Cui, Yaofei Sun

**Affiliations:** ^1^ Department of Urology, Weifang People’s Hospital, Weifang, Shandong, China; ^2^ Department of Nuclear Medicine, Weifang People’sHospital, Weifang, Shandong, China; ^3^ School of Clinical Medicine, Shandong Second Medical University, Weifang, China

**Keywords:** renal cell carcinoma, pregnancy, MIT family translocation, transcription factor E3, transcription factor EB

## Abstract

MiT family translocation renal cell carcinomas (tRCCs) primarily include Xp11.2/transcription factor E3 (TFE3) gene fusion-associated renal cell carcinoma (Xp11.2 tRCC) and t(6;11)/TFEB gene fusion-associated RCC. Clinical cases of these carcinomas are rare. Fluorescence *in situ* hybridization can be used to identify the type, but there are no standard diagnostic and treatment methods available, and the prognosis remains controversial. Herein, we present a case of a patient with Xp11.2 tRCC at 29 weeks of gestation. The baby was successfully delivered, and radical surgery was performed for renal cancer at the same time. This is a unique and extremely rare case. We have described the case and performed a literature review to report the progress of current research on the treatment and prognosis of pregnant patients with Xp11.2/TFE3 translocation renal cell carcinoma. This study aims to contribute to improving the diagnosis and treatment of Xp11.2 tRCC in pregnant patients.

## 1 Introduction

Renal cancer is the third most common tumor in the urinary system, ranking 12th among the most common cancers worldwide. It accounts for 3% of adult malignant tumors, with an incidence twice as high in men as in women (1). Cases of malignant tumors during pregnancy are rarely reported. Approximately 1 in 1,000 pregnant women are diagnosed with malignant tumors before delivery (2). Whether it is the initial detection of a malignant tumor in a pregnant patient or the occurrence of pregnancy during the course of treatment for a malignant tumor, it presents a highly challenging issue in clinical diagnosis and management.

Malignant tumors can develop during pregnancy due to established risk factors such as family genetics, smoking, obesity, multiple pregnancies, chronic kidney disease, hypertension, diabetes mellitus, antihypertensive medications, environmental influences, and poor diet. Additionally, high estrogen levels, prolonged high-fat diets, and a sedentary lifestyle can contribute to malignancy (3). MiT family translocation renal cell carcinoma (RCC) is rarely reported. Specific typing of the MiT family can be identified using fluorescence *in situ* hybridization (FISH) to evaluate the prognostic and survival model of the disease.

Xp11.2 tRCC is characterized by papillary growth, large nucleoli, eosinophilic or hyaline cytoplasm with numerous psammoma bodies, and vesicular or other structures similar to other RCCs. Compared with common RCC, Xp11.2 tRCC is more aggressive and has a poorer prognosis; hence, regular follow-up and early review post-surgery are essential. For pregnant patients with Xp11.2 tRCC, the timing of surgery should be carefully planned, and an individualized treatment plan can be devised based on genetic testing results post-surgery. A comprehensive review of the literature suggests that the combination of targeted drugs and immune checkpoint inhibitors (ICIs) may represent a more effective strategy for cancer treatment.

## 2 Case report

**Figure 1:**
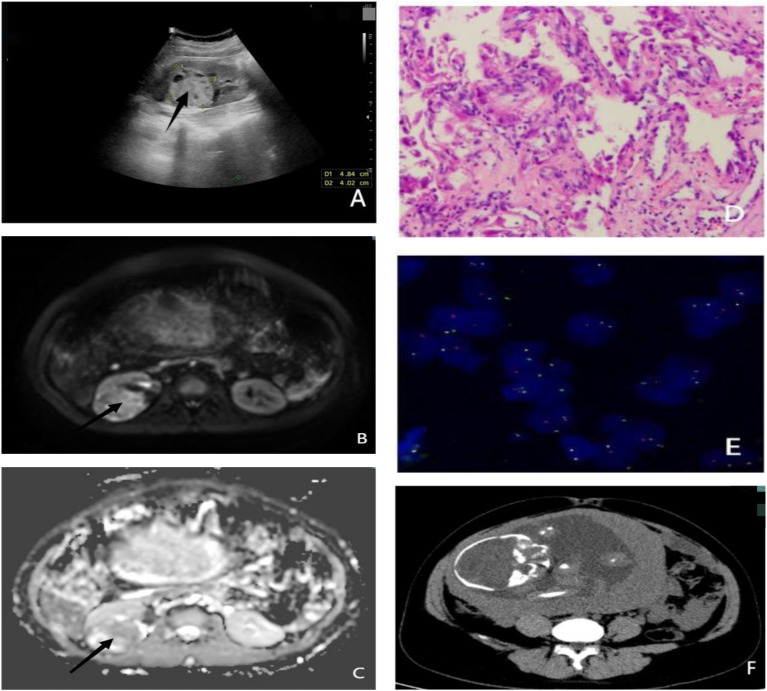
**(A)** Ultrasound showing a hyperechoic cystic-solid tumor of the right kidney measuring 4.8 × 4.0 cm. **(B)** MRI ADC sequence of renal tumor. **(C)** MRI DWI sequence of renal tumor. **(D)** Pathological section: cytoplasmic hyaline in most of the cells, eosinophilic nucleoli in a few cells, and gravel body-like calcifications. **(E)** TFE3 (Xp11.2) gene breakage detection probe revealing broken TFE3 (the ratio of break signals is 43%, exceeding the threshold of 10%). **(F)** Lower abdomen CT showing the 29-week fetal morphology. ADC, apparent diffusion coefficient; DWI, diffusion-weighted imaging.

A 28-year-old married woman was admitted to the hospital with a complaint of hematuria for 1 day at 29 weeks of gestation. The patient reported no distinct triggers or causes for the hematuria. She experienced blood clots, dysuria, and urinary tract irritation symptoms. Prior to admission, she did not have lumbar pain, loss of appetite, fatigue, lethargy, or other systemic symptoms. A lower abdominal computed tomography (CT) scan conducted on September 2, 2023, revealed the following:

1. Mixed density foci in the right kidney, excluding the possibility of space-occupying lesions, suggesting the need for further examination;
2. Presence of blood accumulation in the bladder; and
3. Intrauterine fetal shadow (**Figure 1F**).

After a comprehensive evaluation, the patient was admitted to our Urology Medical Center. The patient’s history was as follows.

Upon admission, physical examination revealed the following: temperature, 36.6°C; pulse, 85 beats/min; respiration, 20 beats/min; and blood pressure, 130/70 mmHg. There was no underlying disease, no obvious swelling on the face and lower limbs, no percussion pain, and no pressure pain in both kidneys.

A dual renal ultrasound revealed a 4.8 × 4.0 cm area of high echoes in the right kidney with clear boundaries. Several small echoes were detected within this area, suggesting a cystic-solid lesion of the right kidney (**Figure 1A**). Dual renal magnetic resonance (MR) examination scanning confirmed the lesion (**Figures 1B, C**).

An evaluation of her pregnancy status revealed a hemoglobin level of 124 g/L. Given the complexity of her condition and the necessity of surgery, a preoperative consultation was conducted with the entire hospital team to determine the optimal timing and plan for the surgery.

### 2.1 Preliminary diagnosis

A malignant tumor of the right kidney (cT3aNxMx) at 29 weeks of pregnancy was found.

The procedure was conducted in two stages. Initially, the infant was delivered via cesarean section by the obstetrician and gynecologist, followed by a laparoscopic radical resection of the right kidney performed by a team of urologists. The operation yielded successful results.

### 2.2 Postoperative pathological diagnosis

Postoperative pathological examination revealed resection of the right kidney along with perirenal fat measuring 13 cm × 9 cm × 4 cm. The renal fat capsule was easily peeled. A mass measuring 4.5 cm × 4.5 cm × 1.5 cm was observed in the kidney. The section appeared grayish red and grayish yellow, was brittle, and had a close relationship with the renal peritoneum. The mass involved the renal pelvis but did not affect the renal sinuses. Additionally, a section of the ureter was involved, and a long lymph node measuring 1.5 cm was detected around the renal hilum.

### 2.3 Pathological results

The tumor in the right kidney was papillary. The dimensions of the renal tumor measured 4.5 cm × 4.5 cm × 1.5 cm. Most renal tumor cells exhibit clear cytoplasm, with a few containing eosinophilic nuclei and granular calcifications. The tumor was classified as WHO/International Society of Urological Pathology (ISUP) grade III. It involved the mucosa of the renal pelvis, renal peritoneum, and perinephric fat, with embolisms in the choledochotomy. No clear nerve invasion was observed. Metastatic cancer was found in the perirenal lymph nodes (1/2), and metastasis was also observed in the lymph nodes of the renal hilum (1/2). The ureter and blood vessel breaks were disrupted.

MiT family translocation RCC was diagnosed based on clinical and immunohistochemical analysis. The FISH test, performed using a transcription factor E3 (TFE3) (Xp11.2) gene break detection probe (**Figures 1D, E**), indicated that the ratio of the TFE3 break signal was 43%, exceeding the threshold value of 10% (4, 5), which suggests TFE3 breaks had occurred.

Pathological staging based on the American Joint Committee on Cancer (AJCC) 8th edition classified the tumor as pT3aN1Mx.

### 2.4 Postoperative treatment

Postoperatively, pembrolizumab 200 mg was administered intravenously once every 3 weeks. This treatment has been maintained for 1 year. No adverse immune-related events occurred, and no signs of local recurrence or systemic metastasis were found. Therefore, the therapeutic effect has been highly favorable.

## 3 Discussion

The diagnosis of RCC during pregnancy is typically incidental and often discovered during routine obstetric examinations. Khaled and Hussein (6) reported clinical symptoms such as low back pain (50%) and visible hematuria (47%) in 105 pregnant patients with RCC. Other rare symptoms include hemorrhagic shock from tumor rupture, hypercalcemia, anemia, fever, and weight loss.

When selecting imaging modalities during pregnancy, ultrasound and MRI are preferred due to their lack of radiation exposure effects on the fetus. In emergencies involving the mother, low-dose CT and nuclear imaging may be used; however, cumulative radiation exposure should not exceed 100 mGy (7). Some studies suggest minimal fetal teratogenicity when CT is performed within the first 2 weeks of gestation. Additionally, in early gestation (≤14 weeks), low doses (>60 mGy) may result in severe intellectual disability, and doses >200 mGy could lead to head deformities in infants (3).

Recently, we reviewed a large body of documents and found that RCC cases during pregnancy are extremely rare, with no standardized treatment protocols. Surgery remains the primary treatment modality. Ethical considerations are crucial, particularly in balancing the physical and mental wellbeing of the pregnant woman with the health of the unborn child. B. Chys et al. (8) emphasized the importance of addressing these ethical issues, specifically in determining whether priority should be given to addressing the physical and mental wellbeing of pregnant women or to ensuring the health of the unborn child.

Managing RCC in pregnancy is of significant societal and familial concern, necessitating collaboration between the departments of urology, obstetrics, anesthesiology, neonatology, and imaging to develop optimal treatment plans. Individualized treatment plans for pregnant women should consider factors such as gestational age, clinical symptoms, tumor location, and size. Effective communication with patients and their families is essential to ensure they understand the benefits and potential drawbacks of the treatment plan.

Considering the main factors influencing the timing of surgery, including gestational age, health status, tumor size, location, and progression, the current consensus among researchers suggests that termination of pregnancy should be avoided whenever possible. Efforts should be made to ensure fetal maturity unless the tumor has advanced to a metastatic stage (9).

For pregnant patients with combined malignant tumors, the timing of surgical treatment is crucial and depends on gestational age. If RCC is diagnosed during early pregnancy (≤14 weeks), immediate surgical intervention is recommended despite the risk of miscarriage. When diagnosed during mid-pregnancy (14–27 weeks), surgery is not recommended due to the risk of uterine contractions, fetal distress, and preterm delivery. Therefore, it is advisable to delay surgery until 28 weeks, at which point a planned cesarean section can be performed simultaneously with renal carcinoma surgery. If RCC is diagnosed during the late stage of pregnancy (28–42 weeks), it is recommended to perform a cesarean section and renal carcinoma surgery simultaneously (10).

Y. Qu and M.J. Magers (11, 12) reported that MiT family translocation RCC accounts for approximately 1%-4% of adult RCCs, with Xp11.2 tRCC comprising more than 90% of MiT translocation RCCs. Additionally, Xp11.2 tRCC accounts for approximately 40% of RCCs in children and 1.6%–4% in adults. The microphthalmia transcription factor family includes microphthalmia transcription factor, transcription factor EB (TFEB), TFE3, and transcription factor EC. Among the transcription factors associated with RCC, TFEB (t (6, 10) translocation/TFEB gene fusion-associated RCC) and TFE3 (Xp11.2 translocation/TFE3 gene fusion-associated RCC/Xp11.2 tRCC) are categorized as MiT family translocation RCC (13).

Regarding pathogenesis, although they exhibit different clinical features, histological patterns, immunohistochemistry, and molecular genetics, both types of RCC occur due to chromosomal translocations that form corresponding chimeric proteins, which help express transcription factors EB and E3, thereby promoting tumor formation.

The TFE3 gene is situated in subband 2 of Zone 1 on the short arm of the X chromosome. Chromosomal fusion and rearrangement with other genes lead to the formation of different gene subtypes of Xp11.2 tRCC. Chromosomal rearrangement is primarily balanced by chromosomal translocation. For example, if the TFE3 gene at Xp11.2 is broken, it balances with another broken chromosome. Another form of rearrangement includes interbranchial inversion of the non-POU domain-containing octomer-binding protein (NONO)-TFE3 and intrabranchial inversion of RBM10-TFE3 and GRIPAP1-TFE3 (14–16).

High-throughput sequencing-based detection has identified various fusion genes of Xp11.2 tRCC and TFE3, including *PRCC*, *LUC7L3*, *SFPQ*, *MATR3*, *NONO*, *RBM10*, *CLTC*, *GRIPAP1*, *FUBP1*, *PARP14*, *ARID1B*, *DVL2*, *EWSR1*, *KHSRP*, *MED15*, *KAT6A*, *NEAT1*, *SETD1B*, and *ASPL*. Additionally, genes on chromosomes 3, 3q23, 10, and 10q23 have been detected (4, 17–20). Many prerequisites for gene fusion are established after the breaking of double-stranded DNA; therefore, studies have focused on factors related to the breaking of the Xp11.2 tRCC TFE3 gene to find breakthroughs at the gene level for treating this carcinoma.

In addition to presenting the diagnosis and treatment of the patient, we provide a summary and overview of surgical and follow-up treatment strategies suitable for Xp11.2 tRCC. In the era of targeted and immunotherapy, significant experience has been accumulated in treating advanced/metastatic MiT family translocation RCC using a combination of drugs and multicenter studies. These drugs, including tyrosine kinase inhibitors (TKIs), mammalian target of rapamycin (mTOR) inhibitors, and vascular endothelial growth factor receptors (VEGFRs), target the VEGF and mTOR pathways. Additionally, ICIs act by normalizing the immune response, downregulating programmed cell death protein 1 (PD-1) expression in T cells to ameliorate the inhibitory effect of PD-1 on T cells, or blocking the PD-1/PD-L1 binding to inhibit the PD-1 pathway.

Y. Komai and T. Nelius demonstrated that advanced/metastatic MiT family translocation RCC does not respond to cytokines such as interferon-α and interleukin-2 (21, 22). Limited efficacy of VEGFR has also been reported, with progression-free survival in patients with Xp11.2 tRCC usually less than 1 year. However, the combination of VEGFRs and TKIs is effective against Xp11.2 tRCC. K. Nishimura et al. (23, 24) used axitinib against advanced/metastatic Xp11.2 tRCC and demonstrated its significant efficacy as evidenced by the reduction of metastatic foci. Many researchers (24, 25) have reported that the combination of sunitinib and axitinib may prolong the progression-free survival of these patients. Cristian Solano et al. (26) confirmed the effectiveness of pazopanib in patients with advanced/metastatic Xp11.2 tRCC. Hyun Jung Lee et al. (27) confirmed the efficacy of combining mTOR inhibitors and PD-L1 blockers against Xp11.2 tRCC by determining the relationship between TFE3 and PD-L1 expression and immune cell proliferation. Thus, the combination of mTOR-C1 inhibitors, such as everolimus and sirolimus, and PD-L1 inhibitors may be clinically beneficial in treating patients with MiT RCC when first-line TKI therapy fails.

According to Eric C. Kauffman et al. (23, 24), MiT-RCC-regulated key pathways may be related to levels of cell cycle proteins including transforming growth factor-β, MET tyrosine kinase, ETS transcription factor, E-cadherin, insulin receptor, and folliculin (28). The points of association between the proteins of these pathways are regulated by Akt. mTOR-C1 is a protein complex in the AKT-regulated pathway associated with TFE3 expression, leading to renal tumor carcinogenesis. Resistance to mTOR-C1 inhibitors due to the presence of mTOR-C2 protein complex feedback loops has been observed during clinical use. Eric C. Kauffman (29, 30) showed that AZD8055/Ku0063794, a dual mTOR C1/C2 inhibitor, was more effective in Xp11.2 tRCC treatment, and its clinical trial results showed no significant cytotoxic side effects, representing a promising breakthrough in Xp11.2 tRCC treatment.

Immune checkpoints, an important component of the immune system, are crucial for preventing autoimmune diseases. However, the efficacy of ICIs against tumor immune escape varies depending on PD-L1 levels. Nevertheless, ICIs are generally effective against tumors. In 2010, T.K. Choueiri and G.G. Malouf found that most patients with Xp11.2 tRCC exhibited an immune escape microenvironment characterized by low PD-L1 levels and CD8+ T-cell infiltration in the tumor stroma and that single-agent PD-1/PD-L1 blockers could not sufficiently improve the prognosis of these patients (24, 31). Recently, ICI analogs such as nivolumab and ibritumomab have shown efficacy against Xp11.2 tRCC (32, 33).

Xieqiao Yan et al. (34) found that in patients with advanced/metastatic Xp11.2 tRCC treated with VEGFR-TKI (axitinib) and a PD-1 inhibitor (pembrolizumab) as first-line therapy, the treatment was effective, with a median progression-free survival of more than 16.6 months and a median overall survival of more than 25.6 months, with the disease remaining progression-free. Therefore, target-immunity combination drugs (e.g., ICI/VEGFR-TKI) can induce neoantigens and lymphocyte aggregation in the tumor microenvironment, especially in tumors with high expression of neovascularization, intercellular stroma, and proliferative gene signals, effectively mediating immunogenic cell death and exhibiting considerable therapeutic efficacy. This treatment strategy, however, should be avoided during pregnancy and lactation.

The postoperative pathology of the present patient confirmed stage III renal cancer (pT3N1M0). However, there is no uniform treatment plan for the follow-up treatment of locally progressive Xp11.2 tRCC. Patients with clinical Xp11.2 tRCC show considerable variability in response to targeted immunotherapy drugs. Genetic testing may guide the precise selection of drugs and individualized treatment.

According to the National Comprehensive Cancer Network (NCCN) guidelines for cT1a stage (Xp11.2 tRCC confirmed by postoperative pathology) renal tumors, renal-conserving surgery followed by postoperative follow-up according to the stage I renal cancer follow-up standard is feasible. For cT1b~cT2 stage (Xp11.2 tRCC confirmed by postoperative pathology) renal tumors, renal-conserving or radical resection surgery is feasible, followed by renal-conserving or radical resection surgery within a limited period after the initial surgery if the patient or family consents. After radical nephrectomy, follow-up must adhere to the criteria for stage II renal cancer. For locally progressive renal cancer (postoperative pathological confirmation of Xp11.2 tRCC), radical nephrectomy is feasible, followed by targeted or immunotherapy as recommended, with follow-up adhering to the criteria for stage III renal cancer.

## 4 Conclusion

Xp11.2 tRCC is a rare subtype of RCC with an unclear pathogenic mechanism. The disease can be diagnosed by FISH to clarify its pathological subtype. There is no standardized follow-up treatment plan available for this tumor. For pregnant patients with Xp11.2 tRCC, the timing of surgery should be carefully planned, and an individualized treatment plan can be devised based on genetic testing results after surgery. The combination of targeted drugs and ICIs may offer a more effective therapeutic strategy against this type of cancer.

## Data availability statement

The original contributions presented in the study are included in the article/supplementary material. Further inquiries can be directed to the corresponding author.

## Ethics statement

Ethical approval was not required for the study involving humans in accordance with the local legislation and institutional requirements. Written informed consent to participate in this study was not required from the participants or the participants’ legal guardians/next of kin in accordance with the national legislation and the institutional requirements. Written informed consent was obtained from the individual(s) for the publication of any potentially identifiable images or data included in this article.

## Author contributions

YW: Writing – original draft, Writing – review & editing. XG: Conceptualization, Investigation, Writing – review & editing. ZM: Supervision, Writing – review & editing. YC: Supervision, Writing – review & editing. YS: Supervision, Writing – review & editing.
